# Genomic Analysis of Sarda Sheep Raised at Diverse Temperatures Highlights Several Genes Involved in Adaptations to the Environment and Heat Stress Response

**DOI:** 10.3390/ani14243585

**Published:** 2024-12-12

**Authors:** Giustino Gaspa, Alberto Cesarani, Alfredo Pauciullo, Ilaria Peana, Nicolò P. P. Macciotta

**Affiliations:** 1 Department of Agricultural, Forest and Food Science, University of Torino, 10124 Torino, Italy; alfredo.pauciullo@unito.it; 2 Department of Agriculture, University of Sassari, 07100 Sassari, Italy; acesarani@uniss.it (A.C.); macciott@uniss.it (N.P.P.M.); 3 Department of Animal and Dairy Science, University of Georgia, Athens, GA 30602, USA; 4 Servizio Agrometeorologico Regionale per la Sardegna (ARPAS), 07100 Sassari, Italy; ipeana@arpa.sardegna.it

**Keywords:** genomic technologies, climate change, fixation index, selection signatures

## Abstract

Livestock expresses complex traits influenced by several factors. The response of animals to variations in climatic factors, such as increases in temperature, may induce heat stress conditions. In this study, animals living at different temperatures were compared using the genome-wide Wright fixation index (*F_ST_*). A total of 825 genotypes of Sarda breed ewes were divided into two groups based on the flocks’ average temperature over a 20-year period to compute the *F_ST_*: 395 and 430 sheep were represented in colder and hotter groups, respectively. After LOWESS regression and CONTROL CHART application, 623 significant markers and 97 selection signatures were found. A total of 280 positional candidate genes were retrieved from a public database. Among these genomic regions, we found 51 annotated genes previously associated with heat stress/tolerance in ruminants (*FCGR1A*, *MDH1*, *UGP2*, *MYO1G*, and *HSPB3*), as well as immune response and cellular mechanisms related to how animals cope with thermal stress (*RIPK1*, *SERPINB1*, *SERPINB9*, and *PELI1*). Moreover, other genes were associated with milk fat (*SCD*, *HERC3*, *SCFD2*, and *CHUK*), body weight, body fat, and intramuscular fat composition (*AGPAT2*, *ABCD2*, *MFAP32*, *YTHDC1*, *SIRT3*, *SCD*, and *RNF121*), which might suggest the influence of environmental conditions on the genome of Sarda sheep.

## Simple Summary

In extensive breeding systems, environmental conditions strongly influence animal behavior and production. During the process of evolution, animals tended to adapt their morphology and physiology to environmental conditions, leaving genomic imprints. This adaptation can be traced in the animals’ genomes, relating environmental features to genome-wide differentiation metrics. In this study, using Sarda sheep living at different temperatures as a case study, we compared their genomes to highlight traces of thermal tolerance and adaptation.

## 1. Introduction

Livestock production is affected by complex traits that can be modeled according to several factors (e.g., genetics, climate, management, and nutrition). It is well known that livestock production and health are affected by environmental conditions. Indeed, environmental adaptation is an important evolutionary feature among animals [1,2]. Thanks to their homoeothermic feature, mammals are able to cope with most extreme environments, distinguished by different temperatures [3]. The process of evolution causes changes in animal genomes, exploiting variations in their morphology (e.g., body size, skin, wool, and fat thickness) and physiological traits (e.g., fat metabolism) in order to adapt to different climatic conditions. Although livestock have adapted to live and produce in different environmental conditions [4], some factors can still have detrimental effects on production, growth, or fitness traits. The response of animals to variations in climatic factors, such as increases in temperature, may induce heat stress [5,6]. The negative effect of temperature, measured using the temperature–humidity index (THI), on productive or reproductive traits, has been the object of several studies in both cattle [7,8,9,10] and small ruminants [11,12,13]. Thermic stress may potentially worsen—even in temperate zones—due to the emerging local modifications of weather associated with climate change [14]. Sheep raised under adverse meteorological conditions face heat stress, which can alter their behaviors. Moreover, physiological changes due to harsh environments can negatively impact reproduction, milk production, and composition [15,16].

Climate change represents a major challenge that governments, research institutions, and agribusiness stakeholders must face in the very near future. In this respect, it is of crucial importance to identify genotypes that enable the production of food under changing environmental conditions. To date, landscape genetics approaches have investigated genome changes driven by environmental conditions, analyzing the effects of temperature, rainfall, altitude, and other environmental features on the genotype distribution using an integrative framework that combines landscape ecology, population genetics, and molecular markers [17,18]. This approach aims to understand how environmental factors modify gene flow and animal genotypes [19] and predict the spread of diseases (or disease vectors) and harmful species [20].

Burrow [21] pointed out that breeding schemes must account for the environment in which animals are raised. The large availability of genomic data has allowed for comparison of divergent breeds and the identification of selection signatures for artificial selection [22] or environmental adaptation [23,24]. Moreover, by studying indigenous breeds living in hot and arid environments, complex gene networks, which mediate the capabilities of sheep and goats to face environmental gradients, have been hypothesized [25,26].

In our view, sheep breeds from southern Europe may represent an interesting model by which to study the effects of environmental adaptation using SNP data, due to the low impact of artificial selection and predominantly extensive farming system [27]. To minimize confounding factors (e.g., the difference in demographic history that occurs when a joint analysis of multiple breeds is carried out) as much as possible, in our study, only one breed was investigated with the aim of detecting the genomic signatures of temperature gradients.

The Sarda breed is autochthonous in Sardinia but is also raised in Central and Southern Italy. Sarda sheep account for about 80% of Italian dairy sheep and 43% of Italian ovine stock [28,29]. These animals are well adapted to different types of farming systems, ranging from extensive to semi-intensive pasture-based farming. Sarda sheep are characterized by their small–medium size with white fleece and polledness in both sexes. They are generally selected for their milk quantity and resistance to scrapie [30]. The genetic merit of their breeding stock varies according to the production system in operation, due to limited ram exchanges and large variability among geographic zones. Their milk is fully transformed into cheese, with a production of 60,000 tons/year [31].

The aim of this study was to identify traces of environmental adaptation in the Sarda sheep genome by comparing two groups of animals. The Sarda population provides a good example of sheep that have adapted to the Mediterranean climate, and the test groups were defined according to the historical average temperatures of the zones where they are raised: one group living in the hot areas and another in the cold areas of the second-largest Mediterranean island.

## 2. Materials and Methods

### 2.1. Animal and Genotypic Data

A total of 825 Sarda breed dairy ewes were genotyped using the Infinium Ovine SNP50 v1 BeadChip (Illumina Inc., San Diego, CA, USA), containing 54,241 markers. Quality control was carried out using PLINK v1.9 [32] on both subjects and SNPs. No animals were discarded, according to a call rate < 0.95. Subsequently, SNPs were filtered based on the call rate (>0.975), deviation from the Hardy–Weinberg equilibrium (*p*-value < 0.00001), and minor allele frequency (>0.02), and, ultimately, 45,947 markers were used.

The ewes, farmed in 46 different flocks (Supplementary Figure S1), were allocated into two groups—“cold environment” (CE, 395 ewes) and “hot environment” (HE, 430 ewes)—according to the average maximum temperature over a 20-year period recorded by a network of 39 out of the 60 weather stations operated by the regional department of the environmental protection agency (ARPAS, http://www.sardegnaambiente.it/arpas/ accessed on 10 July 2024) of Sardinia. A neighborhood criterion was adopted for matching the genotypic record to CE or HE class: 50 km was the maximum distance from the flocks that allowed us to consider them belonging to a particular weather station. The breaking temperature was fixed at 21 °C. The animals were selected from large numbers of flocks in order to be largely representative of herd book variability. The 825 dairy ewes were the offspring of 499 rams, with 2.1 ± 1.7 ewes served by each ram on average. The Sarda samples were plotted against the first two principal coordinates of the multidimensional scaling of genotype data using the --mds-plot flag of PLINK v1.9 [32] (see Supplementary Figure S2).

### 2.2. F_ST_ Evaluation

Wright’s fixation index (*F_ST_*) was used following the metrics proposed by Nei [33] and implemented via an in-house Python script. Subsequently, a locally weighted scatterplot smoothing regression (LOWESS) was applied using PROC LOESS in the SAS software 9.2 (SAS Institute, Cary, NC, USA) according to [34] (the smoothing parameters are reported in Table S1). The smoothing parameters were chosen in such a way that each window interval always included 20 SNPs. The chromosome-wide smoothing parameters were computed as 20/number of SNPs. The control chart of PROC SHEWHARD in SAS was used to identify outlier markers that exceeded three standard deviations from the chromosomic mean value, according to Sorbolini et al. [35] (see Supplementary Files for full scripts). The Manhattan plot of LOESS-predicted *F_ST_* was prepared using the qqman package (v. 0.1.9) in R (v. 4.3.1).

### 2.3. Gene Annotation Analysis

Annotated genes in genomic regions corresponding to the *F_ST_* outlier signals associated with thermal gradients were downloaded from the National Center for Biotechnology Information database (www.ncbi.nlm.nih.gov, accessed on 15 July 2024) using the Ovis_aries_rambouillet.ARS-UI_Ramb_v2.0.112.gtf assembly file and retrieved with the GALLO package (v. 1.5) in R [36] via the functions import_gff_gtf() and find_genes_qtls_around_markers(). An interval of 250 kb upstream and downstream from each significant marker was considered. Additionally, from the gene list, genes previously associated with environmental adaptation were sought from public databases. Gene enrichment analysis was conducted using the R package gprofiler2 (v 0.2.3) [37], with *homo sapiens* set as the reference model. A co-expression pattern network was also analyzed (https://string-db.org/ accessed on 10 September 2024).

## 3. Results

### 3.1. F_ST_ Outliers

**Figure 1:**
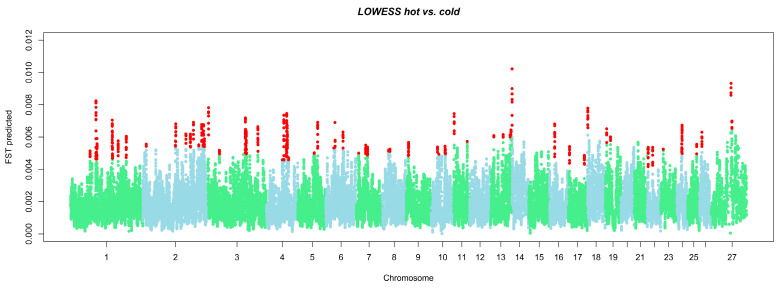
Smoothed *F*_ST_ values predicted with LOWESS across the 27 chromosomes. Red dots represent outlier markers, which exceeded the three standard deviations from mean value (X chromosome is labelled with 27). Light-green and blue dots represent odd and even chromosomes, respectively.

In Figure 1, smoothed *F_ST_* values predicted with LOWESS are reported for each of the 27 ovine chromosomes, highlighting the upper signals: 623 SNPs resulted as outliers in the *F_ST_* values and were grouped into 97 selection signatures based on the CC approach (Supplementary Table S1). OAR1 was the chromosome with the highest number of outliers (83), while OAR12 showed the lowest number of SNPs (5). The smoothed *F_ST_* values ranged from 0. 0005 to 0.01. On average, the smoothed *F_ST_* values were lower than non-smoothed ones and lower than those retrievable from the literature on sheep breed comparison [38].

### 3.2. Positional Candidate Genes

From the outlier signals detected via *F_ST_* analysis (Supplementary Table S2), 280 positional annotate genes in 27 chromosomes were retrieved using the GALLO software package (v. 1.5) within a 500 kb-interval from the peak SNPs. Annotation details are reported in Supplementary Table S3. Around the declared significant markers, 51 genes in 17/27 sheep chromosomes have been previously associated with several phenotypes in domestic ruminants using different approaches (e.g., thermal tolerance, thermal stress, adaptation to extreme conditions, milk yield and fat composition, body fat composition, and fatty acid metabolism). Detailed descriptions of retrieved genes, their roles, and a full bibliography are all provided in Supplementary Table S4.

Table 1 shows the most meaningful selection signatures in *OAR1*, *OAR2*, *OAR3*, *OAR4*, *OAR5*, *OAR6*, *OAR8*, *OAR11*, *OAR15*, *OAR16*, *OAR20*, and *OAR22.* A total of 23 genes found in this study were previously putatively related to heat stress in ruminant GWASs for heat stress (*FCGR1A*, *MRPL9*, *SERPINE2*, *MDH1 UGP2*, *RIPK1*, *SERPINB9*), in candidate gene studies (*HSPB3*, *SOCS3*), or were differently expressed genes (DEGs) in RNA-seq experiments with animals subjected to heat stress condition (*TDRKH*, *DNER*, *UGP2*, *MYO1G*, *SORL1*, *SERPINB1*, *HIF1AN*). Besides the genes directly involved with heat stress, Table 1 also highlights genes that were found in the literature as selection signatures of environmental adaptation, defined using climatic or other environmental variables (*NMUR1*, *PDE6D*, *COPS7B*, *STK17A*, *MFAP3*, *COMMD8*, *SCFD2*, *SNX3*, *RNF121*).

The other 32 genes highlighted in our study have been previously associated with phenotypes that might be indirect indicators of climatic adaptation. In particular, 25 genes were previously found to be associated with physiological traits that regulate metabolism, the mobilization of energy, and increased metabolic heat body: body weight (*PHGDH*, *SGCB*, and *TPM1*), skeletal muscle development in sheep and cattle (*SCAMP1*, *HIF1AN*), fatness and fat deposition (*RORC*, *AGPAT2*, *ABCD2*, *MFAP32*, *YTHDC1*, *RNF121*, *SIRT3*, *SCD*, *RNF121*, *LYRM1*), intramuscular fatty acid composition (*AGPAT2*, *ABCD2*, *MFAP3*, *SCD*), and fat-type tail in sheep (*COPS7B*, *PDE6D*, and *USPL1*). They may be indirectly linked to heat tolerance. They are also involved in cell molecular mechanisms related to wool bulb regression and regeneration in sheep (*S100A11*), ultraviolet radiation resistance (*EDEM1*), and immune response (*FNDC3B*, *PELI1*) (Table 2). Finally, 11 genes were found to be associated with productive traits, such as amount of milk (*HERC3*, *SCFD2*, *CHUK*, *EEF2K)*, milk fat (*SPATA16*, *GALNT10*, *SCD*, *SEC31B*, *ZP2*), or immune response (*PELI1*, *HERC3*) (Table 3).

Eight positional candidate genes highlighted in our study (*MFAP3*, *RNF121*, *HIF1AN*, *PELI1*, *PDE6D*, *COPS7B*, *SCFD2*, *SCD*) were linked to more than one phenotypic or environmental trait and identified in more than one species (recurrent genes in Table 1, Table 2 and Table 3).

Gene enrichment analysis was conducted in order to profile the 51 genes reported in Table 1, Table 2 and Table 3, highlighting a significant intersection among these genes relative to GO molecular functions, cellular components, and binding proteins (Table 4 and Table S5). The GO:0019771 (high-affinity IgG receptor activity) was related to immune response (*FCGR1A*). GO:0030060 and GO:0030060 (*MDH1* and *PHGDH* genes; malate dehydrogenase activity) exert an important metabolic role in energy production. GO related to the inhibition of molecular function or enzyme activity (GO:0004857, GO:0140678) presented the same gene intersection (*SERPINE2*, *PDE6D*, *SOCS3*, *SORL1*, *SERPINB9*, *SEPINB1*) as the GO for peptidase inhibitors and regulations (GO:0004866, GO:0030414), which relate to interaction among the *SERPINE2*, *SORL1*, *SERPINB9*, and *SERPINB1* genes. These are protease inhibitors that may protect cells from damage by inhibiting excessive proteolysis during inflammation conditions [98]. The GO-associated cellular components were mainly related to endo- and extra-cellular vesicle trafficking; in particular, GO:0031410 (cytoplasmic vesicle) and GO:0097708 (intracellular vesicle) presented a significant intersection for 11 genes (*S100A11*, *SERPINE2*, *UGP2*, *MDH1*, *MYO1G*, *SNX3*, *SORL1*, *PTPRJ*, *SERPINB9*, *SERPINB1*, *PHGDH*). Moreover, the STRING protein–protein interaction network, based on co-expression and co-occurrence in databases or experiments, is shown in Supplementary Figure S3.

## 4. Discussion

Response to heat stress and heat tolerance are generally treated as quantitative traits [58] as they are assumed to have a polygenic background. The determinism of heat tolerance, measured according to the THI, is genetically negatively correlated with production traits—at least in dairy cattle—and improving this feature may lead to detrimental effects on other economic traits [99]. The knowledge of genetic variants associated with heat tolerance may be a first step towards dissecting the genetic architecture of such a complex trait. Several studies investigating the associations between thermal stress and genotypes [100,101,102] have identified genes likely to affect this trait. In our investigation, the use of an *F_ST_*-based approach allowed us to suggest positional candidate genes close to outlier SNP markers. Many of these genes have been already associated with the response to thermal stress (Table 1) or environmental adaptation (Table 2 and Table 3) in the literature.

### 4.1. F_ST_ Outliers and Response to Thermal Stress

In our study, the predicted *F_ST_* values were evaluated in terms of a chromosome-wide distribution, rather than as absolute *F_ST_* values. This is because they were the product of a local regression that emphasizes larger values when the neighboring SNPs also presented high values (probable outlier signals), whereas the same signal was regressed toward the window’s mean if the majority of neighboring *F_ST_* had lower values [35]. Most of the signals were present in the first three sheep chromosomes, reflecting the larger size of the chromosomes. In the following, the most meaningful selection signatures are discussed according to the results presented in Table 1.

In OAR1, we found seven selection signatures (Supplementary Table S2). Two nearby *F_ST_* signals (98.7–101.1 Mb) corresponded to the *Fragment of IgG Receptor Iα* (*FCGR1A*) and *Mitochondrial Ribosomal Protein L9* (*MRPL9*) genes, which have been found to be previously associated with heat stress response in Zebu cattle [39]. Mehla et al. [39] have pointed out that gene expression patterns diverged between animals exposed to heat stress and groups of animals raised under optimal climate conditions. The *FCGR1A* gene was observed to be suppressed at 4 h after heat stress but was induced at 24 h and 48 h into heat recovery.

Ten selection signatures were detected on OAR2, corresponding to 23 genes (Supplementary Tables S3 and S4). Four *F_ST_* signals between 22.6 and 23.3 Mb were further investigated, where we retrieved the *SERPINE2*, *NMUR1*, *COPS7B*, and *PDE6D* genes. Dikmen et al. [41] found that *SERPINE2* explained 3.0% of the variance in the sweating rate. This gene, which has been reported to be associated with sweating rate and thermoregulation in dairy cows during heat stress, produces a proteinase-inhibiting thrombin and urokinase-type plasminogen activator [41]. Furthermore, it is involved in most of the molecular processes linked to the inhibition of peptidase (Table 4, GO:0004857, GO:0140678, GO:0004866, GO:0061135, GO:0051248). The inhibition of thrombin can induce changes in epithelial cells from human eccrine sweat glands [103]. The *Neuromedin U Receptor 1* (*NMUR1*) gene was found to be a signature of positive selection for environmental adaptation in sheep [23]. The *NMUR1* gene encodes for a receptor of *NMU* involved in appetite regulation [104], which is generally suppressed during heat stress. The *COPS7B* and *PDE6D* genes were suggested in our study as positional candidate genes related to adaptation to temperature. These findings are in agreement with Gouveia et al. [42] for *COPS7B* in sheep, whereas no indication was reported for *PDE6D* [42]. Despite there being no evidence directly linking *PDE6D* to heat stress in ruminants, regarding its involvement in stress responses and the regulation of cellular processes, Gouveia et al. [42] proposed *PDE6D* as a positional candidate gene that differentiates Brazilian locally adapted sheep breeds.

In OAR3, six selection signatures were identified (Supplementary Table S2). In the region 44.1–44.5 Mb, five genes were observed as potential candidates (*PEL1*, *VPS54*, *UGP2*, *WDPCP*, *MDH*; see Table S2). Among the genes reported in Table 1, *MDH1* (*Malate Dehydrogenase 1*) is an important metabolic enzyme that catalyzes reactions in the tricarboxylic acid cycle pathway, thus playing a role in energy production [43]. *MDH1*, together with *UDP-Glucose Pyrophosphorylase 2* (*UGP2*) and *Dimethylglycine Dehydrogenase* (*DMGHD*), were hypothesized as candidate genes for heat tolerance by Cheruiyot et al. [43]; they also were found to be overexpressed in liver tissue in an experiment relating heat stress conditions with physiological parameters in dairy cows [45]. These three genes are over-represented in both the present study and the existing literature, as the citrate cycle/tricarboxylic acid cycle pathway is central to mitochondrion energetics and might help to prevent cellular damage during heat stress.

A total of 54 SNPs resulted as outliers for *F_ST_*, defining four selection signatures in OAR4 (28 genes; see Supplementary Tables S1 and S2); in particular, in the range between 78.7 and 79.7 Mb, we retrieved two genes: *Serine*/*Threonine Kinase 17a* (*STK17A*) and *Myosin IG* (*MYO1G*) (Table 1). The *STK17A* gene has been suggested by Yang et al. [50] as being positively selected for high-altitude and arid environment adaptation in Tibetan sheep, given its role as a regulator of cellular reactive oxygen species (ROS)—an important functional activity in the pathway of *Hypoxia-inducible Factor1* (*HIF1*). More interestingly, the subunit 1-α of *H1F* (*HAF1AN,* located in OAR 22: 21.1–21.2 Mb, Table 1) retrieved in our study has been reported to be overexpressed in heat-stressed dairy cattle [105]. The plasmatic *HIF-1α* of heat-stressed cows—indicative of the risk of oxidative stress—showed a higher level under heat stress conditions [105]. The second gene, *MYO1G*, was upregulated in the peripheral blood leukocytes of Zebu cattle exposed to heat stress [46]. In addition, [106] has reported *MYO1G* as a positional candidate in a selection signature emerging from a comparison among African cattle breeds. The *MYO1G* gene is associated with immune response and host defense, playing a specialized role in immune cells (i.e., T cells and leukocytes), where it regulates cell migration and immune synapse formation [107]. The *MYO1G* gene is part of the unconventional myosin family, a group of proteins involved in intracellular transport, cell motility, and cytoskeletal organization. Our enrichment analysis collocated *MYO1G* in several gene intersections for GO related to vesicle, extracellular vesicle, and extracellular exosome (GO:0031982, GO:1903561, GO:0043230, GO:0070062, GO:0070062; Table 4). Unlike conventional myosins, which participate in muscle contraction, unconventional myosins such as *MYO1G* play roles in diverse cellular functions (particularly related to membrane dynamics), and have been associated with actin cytoskeleton remodeling and plasma membrane functions (e.g., cytoskeletal integrity, cell motility and migration, and membrane repair), as well as the regulation of cell stiffness in B-lymphocytes [107]. This latter function is necessary for the recovery of membrane proteins and the prevention of cellular damage. Indeed, heat stress could damage proteins at the cell surface, and *MYO1G* might aid in their internalization and processing via the endosomal system. This is vital for cell survival, as it prevents the accumulation of damaged proteins on the membrane. Heat stress often initiates an immune response, and *MYO1G* could play a role in ensuring that immune cells maintain their ability to move and respond to stress-induced inflammation. Sheep use various mechanisms to adapt to heat stress, such as increasing their respiration rate and changing their body temperature [6]. The *MYO1G* gene, being involved in cellular transport and cytoskeletal interactions, may potentially play a role in maintaining cellular integrity under heat stress conditions [108].

OAR5 presented four selection signatures and five positional candidate genes, as reported in Supplementary Tables S1 and S3 (*NMUR2*, *FAM114A2*, *MFAP3*, *GALNT10*, *HAND1*). Although only *Microfibril Associated Protein* 3 (*MFAP3*) has been significantly enriched under thermal tolerance conditions in local Chinese cattle [48], *NMUR*2 is the second receptor of the aforementioned *NMU*, which has been observed in brain sites that are important for appetite regulation [104].

Interestingly, outlier *F_ST_* signals were retrieved for OAR6 (seven outlier signals and 19 genes), OAR8 (two outliers, seven genes), OAR11 (two outliers, 12 genes), OAR15 (three outliers, 10 genes), OAR16 (three outliers, five genes), OAR20 (one outlier, nine genes), and OAR22 (two outliers, 22 genes). The outlier SNP markers and positional candidates retrieved in these genomic regions are reported in Supplementary Tables S2 and S3. The region between 66.9 and 69.6 Mb, where a gene related to immune response was found (the *COMM Domain Containing 8* (*COMMD8*), is of particular interest. Although there is limited evidence regarding *COMMD8* in the context of heat stress, this gene has been reported, using a landscape genomic approach [49], to be a positional environmental outlier. Indeed, members of the *COMMD* family are involved in regulating *Nuclear Factor k Chain Transcription in B Cells* (*NF-κB*) signaling and copper homeostasis [109], both of which might be influenced by thermal stress. The proper regulation of *NF-κB* is important for managing the inflammatory response and protecting against heat-stress-induced cell apoptosis [110]. The *F_ST_* signals in OAR11 (28.9–29.2) were close to the *SOCS3* gene map (*Suppressor of Cytokine Signaling 3*). *SOCS3* is involved in regulating cytokine signaling pathways, which are a crucial cellular stress response mechanism [111]. Heat stress induces an inflammatory response, and *SOCS3* helps to modulate this response by negatively regulating pro-inflammatory cytokines such as *IL-6* and *TNF-α* [112]. In bovines, *SOCS3* downregulated *GH*-dependent transcriptional activation in response to heat stress [52]. Although the same authors [52] pointed out that heat stress alone was partially responsible for the *SOCS3*-related negative modulation of hepatic GH receptor abundance, a reduction in *GH* levels during thermal stress has also been observed by other authors [6]. In OAR15, the *Sortilin-related Receptor 1* (*SORL1*) gene highlighted in our study has also been shown to be upregulated in the peripheral blood leukocytes of Zebu exposed to heat stress [46]. One of the three *F_ST_* outlier signals in OAR16 (24.8 Mb) mapped close to the positional candidate *Heat Shock Protein Family B (small) Member 3* gene (*HSPB3*). *HSPB3* has been associated with the heat stress response [56]. These authors compared *miRNAs* levels and target genes in two Holstein cattle groups: one heat-stressed and the other not. The *HSPB3* gene was significantly associated with stress exposure. Moreover, other heat-response miRNA showed differences in the induction of their expression levels between buffalo and native cattle [112]. There is no direct link between the *HSPB3* gene and heat stress in ovines; however, heat-shock proteins (*HSPs*) are well known to play a critical role in cellular responses to stress, including thermal stress, across different species [46]. *HSPB3* expression in the muscles of chickens raised in lowland areas varied according to the time of the day, in contrast to highland chickens [113]. These proteins are key players in protecting cells from the damaging effects of heat as they ensure proper protein folding and prevent aggregation. Although *HSPB3* has not been highlighted in the same way for ovine heat stress, it could potentially play a similar protective role in muscle or other tissues as it does in other animals. The more commonly studied *HSP70* and *HSP90* genes have been associated with heat adaptation in domestic ungulates [114].

In OAR20, one selection signature and nine genes were retrieved from a public database (Supplementary Tables S1 and S2). Three interesting positional candidate genes, *RIPK1*, *SERPINB9* and *SERPINB1*, located at 49.6–49.9 Mb, are involved in immune response and will be jointly discussed due to their roles in the negative regulation of the protein metabolic process (Table 4) and because they have been reported to be related to heat stress responses in ruminants. The *Receptor Interacting Serine*/*Threonine Kinase 1* (*RIPK1*) gene is central to cell survival, apoptosis, and necrosis. Under heat stress, cells undergo apoptosis as part of a protective mechanism for removing damaged cells and preventing inflammation due to necrotic cell death [115]. The *RIPK1* gene has been proposed as a positional candidate that would explain beef cattle body temperature during heat stress [57]. The *Serpin Family B Member 9* (*SERPINB9*) gene has also been proposed for this function. These genes code for proteins involved in inflammation and cellular death in response to external stresses that cause tissue damage. Heat stress often triggers oxidative stress, protein misfolding, metabolic alterations, and immune system activation, making genes associated with these processes especially important for adaptation to heat stress [116]. In other mammalian models, under heat shock conditions, *RIPK1* activity helps to balance the decision between cell survival and death [117]. *Serpin Family B Member 1* (*SERPINB1*) is a protease inhibitor that protects cells from proteolytic damage during stress, which has been found to be downregulated in Zebu cattle exposed to heat stress [46]. The same author explained the reduction in the expression of immune response genes as a consequence of reduced metabolic activity and feed intake during heat stress [46].

### 4.2. F_st_ Outliers and Environmental Adaptation

Climate adaptation also has consequences for different livestock features, such as body size and composition, lipid metabolism, and changes in the amount and quality of milk production. Livestock body size is a complex phenotype, affected by several different features, which has undergone severe transformation during evolution: adaptations to the environment are strongly associated with body dimensions in mammals, humans included [3,118,119,120]. In Table 2, the genes found in this study, which have been associated in the existing literature with body size, amount of fat, and lipid metabolism, are reported.

*HAF1AN* was identified as a transcriptional regulator (either in ovine and bovine contexts) in a comparative gene expression study on skeletal muscle *longissimus thoracis* muscle [82], but has also been associated with heat stress, due to its synergistic activation of the expression of *HSP* family members in dairy cattle [105].

As far as wool is concerned, the *S100 Calcium Binding Protein A11* (*S100A11*) gene was mapped in a selection signature highlighted in this study. This gene has been reported to be associated with wool characteristics [61]. These authors observed the overexpression of this gene in the second stage of the life cycle of hair bulbs; the *S100A11* gene was also associated with the inhibition of keratinocyte apoptosis [121].

*EDEM1* is an intriguing gene for environmental adaptation. Indeed, *EDEM1* is associated with lifespan in *Drosophila melanogaster* and *Caenorhabditis elegans* [122]. *EDEM1* levels were reduced in fibroblasts obtained from a dwarf mouse model of longevity. These fibroblasts were more resistant to cell death from stressors such as UV light [76] and have been linked to the youthfulness of skin in humans [75].

*Pellino E3 Ubiquitin Protein Ligase 1* (*PELI1*) and *HECT* and *RLD Domain Containing E3 Ubiquitin Protein Ligase 3* (*HERC3*) are candidate genes associated with immune responses in animals, regulating the inflammatory response initiated by *NF-κB* [123]. Despite there being no direct evidence of the effects of these genes on thermal stress, prolonged exposure to high temperatures has negative consequences on the immune response in animals. Furthermore, Gupta et al. [124] have reported that heat stress causes a decrease in white blood cell count.

Additionally, in the literature, the *Protein Tyrosine Phosphatase*, *Receptor Type J* (*PTPRJ*) gene has been associated with high-altitude adaptation in Cashmere breed goats [73]. As previously mentioned, the tail type has a strong association with climate adaptation, especially in sheep [72]. Taking a *F_ST_*-based approach, Yuan et al. [72] observed a significant signal of the differentiation between thin- and fat-tail sheep near the *Ubiquitin Specific Peptidase Like 1* (*USPL1*) gene, where the latter is known to be an adaptative trait. *COP9S7B* was suggested in this article to be a positional gene that relates to temperature adaptation in sheep, in agreement with [42], but *COP9S7B* has also been highlighted as a selection signature that differentiates fat-tail from non-fat-tail sheep [62].

For many genes found in our study (*RORC*, *AGPAT2*, *AGCD2*, *MFAP3*, *YTHDC1*, *RNF121*, *SIRT3*, *SCD*), genetic associations with body fat composition [59,63,64,65,66,69,74,77] and intramuscular fatty acid metabolism have been reported [78,79,80,81] (Table 2 and Supplementary Table S4); other interesting genes have been associated with milk fat in dairy ruminants (*GALNT10* [85], *CHUK* [90], *SCFD2* [51,88,89], *SEC31B* [94] *ZP2* [95,96], *SCD* [79,81,91,93] and *SPATA16* [84], Table 3). Hormones secreted during stressful situations (e.g., epinephrine) have been observed to induce lipolysis and to increase the amount of NEFAs, and similar mechanisms could be hypothesized for thermal stress. However, ref. [52] has reported that fat mobilization in cows under stress was not as intense as expected.

### 4.3. Heat Stress and Immune Response

Many of the genes listed in Table 1, Table 2 and Table 3 are involved in cellular stress responses, including protein folding (*HSPB3*), immune regulation (*MYOG1*, *SORL1*, *SOCS3*, *SERPINE2*, *CHUK*, *HERC3*, *PELI1*), metabolic adaptation (*SIRT3*, *MDH1*), and apoptosis control (*STK17A*, *RIPK1*, *SERPINB1*, *SERPINB9*). These genes, both individually and collectively, might contribute to the ability of sheep to mitigate the detrimental effects of heat stress. They ensure that cells maintain homeostasis by controlling inflammation, promoting survival pathways, and ensuring proper protein handling during periods of thermal stress [47,52,53,54].

**Figure 2:**
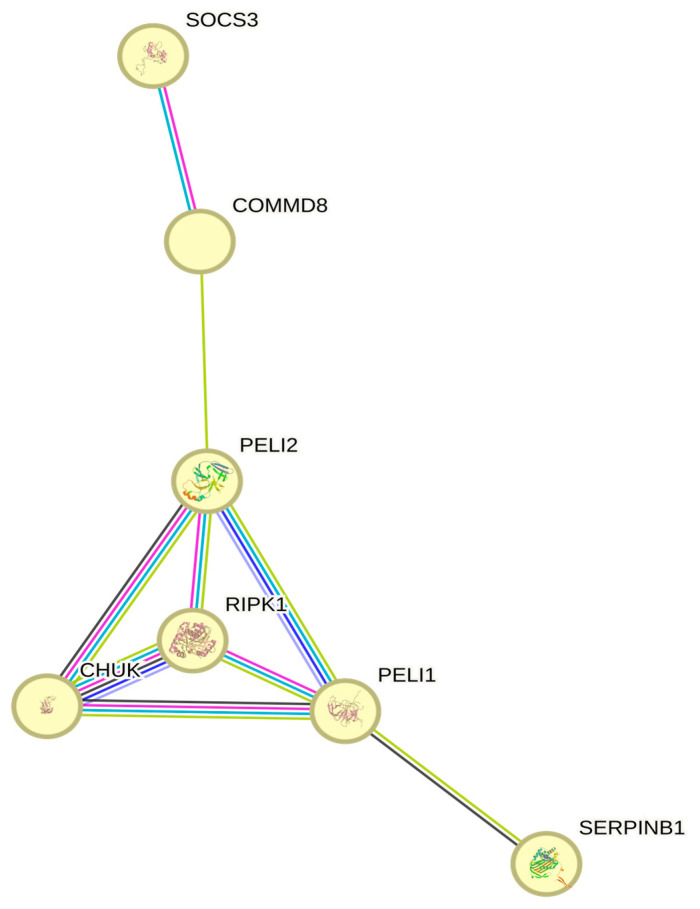
Protein–protein interactions for a subset of genes from the STRING network.

The small network reported in Figure 2, derived from the larger protein–protein interaction network composed of genes retrieved in this study (Supplementary Figure S2), includes the key regulators of inflammatory responses, apoptosis, *NF-κB* signaling, and protease inhibition, all of which are crucial for cells to survive and adapt under heat stress conditions. Heat stress responses can be related to different molecular mechanisms, such as the inflammatory response or the activation of cell death pathways. The *SOCS3* gene helps to regulate cytokine activity, preventing excessive inflammation [110], whereas proteins such as *RIPK1* mediate cell death decisions to ensure the damaged cells are cleared without excessive necrosis, which would lead to inflammation [115]. On the other hand, molecular mechanisms related to survival pathways and protective action might be associated with heat stress responses [117]. *NF-κB* pathway members such as *CHUK* and *COMMD8* activate survival pathways (inducing the expression of heat shock proteins and other protective molecules) [109], while *SERPINB1* prevents protease-induced damage during stress response, making it a key player in cellular homeostasis under heat stress [57]. *Conserved Helix Ubiquitous Kinase* (*CHUK*) is part of the *NF-κB* signaling pathway, which plays a role in responding to stress stimuli; it has also been associated with mammary gland health in dairy sheep [90]. Heat stress can activate *NF-κB* signaling, leading to the expression of *HSP* and inflammatory mediators. This helps cells to cope with the accumulation of damaged proteins and other stress-induced injuries. In cattle, the *NF-κB* pathway was activated under heat stress, enhancing the expression of protective proteins such as *HSP*, which refold damaged proteins and prevent protein aggregation [125].

*PELI* proteins (*PELI1*/*PELI2*) exert functions in the immune signaling pathways, especially in the activation of toll-like receptors (*TLR*) and interleukin-1 (*IL-1*) pathways [126]. These pathways are important for the innate immune response, which is activated during heat stress. *PELI* proteins help to propagate signals that lead to the activation of *NF-κB* which, in turn, induces the expression of heat shock proteins and other stress-response genes [47].

## 5. Conclusions

Heat stress is a well-known problem on dairy farms, due to its negative impacts on milk production. The financial loss related to this stress in the American dairy industry has been estimated at USD 900 million per year (dairybusiness.com, accessed on 10 September 2024). Losses due to climate change and the associated increase in maximum temperatures are expected to be further exacerbated in the future. This problem is particularly acute in subtropical and hot regions where, most of the time, animals are raised in semi-extensive or extensive conditions; in the Mediterranean area, for example, ewes spend most of their time outside, especially in the hot season. In these environments, heat stress leads to decline in both the amount and quality of milk. Our study investigated climate-mediated selection signatures using genome-wide differentiation metrics computed between two groups of dairy sheep reared in the same climatic zone but in areas characterized by different maximum temperatures. The genes highlighted in this study have been previously associated with heat tolerance or adaptation traits in the existing literature. This study suggests the suitability of a simple analytical approach for retrieving positional candidate genes associated with heat stress and other adaptation traits, ultimately arguing for the viability of this model in studying climate adaptation in animals.

## Figures and Tables

**Table 1:** Retrieved genes already associated in the literature with adaptation, heat stress/tolerance, or adaptation traits. The reported genes fell within the region of 250 kb upstream and downstream from *F_ST_* outlier SNPs.

OAR	Position (bp) ^1^	Gene Acronym ^2^	Adaptation Traits	Approaches in Literature	Species	Ref
1	99876667:99912023	*FCGR1A*	Response to heat stress	GWAS	*Zebu*	[39]
1	101684582:101692525	*MRPL9*	Response to heat stress	GWAS	*Zebu*	[39]
1	101694230:101722463	*TDRKH*	Response to heat stress	Gene expression	*Cattle*	[40]
2	226126906:226195781	*SERPINE2*	Response to heat stress	Candidate gene/validation	*Cattle*	[41]
2	233283110:233291092	*NMUR1*	Adaptation to heat stress	Selection signature	*Sheep*	[23]
2	233423272:233484780	*PDE6D* *†	Environmental adaptation	Selection signature	*Sheep*	[42]
2	233485205:233510593	*COPS7B* *†	Environmental adaptation	Selection signature	*Sheep*	[42]
3	44695544:44719947	*MDH1*	Heat stress	GWAS	*Sheep*/*Cattle*	[43,44]
3	44379444:44434924	*UGP2*	Heat stress	GWAS/Gene expression	*Cattle*	[43,45]
4	78286795:78303108	*MYO1G*	Heat stress	Gene expression	*Zebù Cattle*	[46]
4	79132035:79170008	*STK17A*	Adaptation extreme env.	Selection signature	*Sheep*	[47]
5	63118084:63132662	*MFAP3* *	Adaptation to heat stress	Selection signature	*Cattle*	[48]
6	66800148:66815074	*COMMD8*	Adaptation to heat stress	Selection signature	*Swine*	[49]
6	69212902:69607896	*SCFD2* *‡	Environmental adaptation	Selection signature	*Cattle*/*Sheep and Goats*	[50,51]
8	29178799:29228203	*SNX3*	Adaptation to heat stress	Selection signature	*Sheep*	[42]
11	53173956:53178761	*SOCS3*	Heat stress/High altitude	Candidate gene	*Cattle*	[52,53,54]
15	31804101:31966090	*SORL1*	Heat stress	Gene expression	*Zebù Cattle*	[46]
15	50285158.:50365195	*RNF121* *†	Environmental adaptation	Selection signature	*Sheep and Goats*	[55]
16	24828044:24841106	*HSPB3*	Heat stress	Candidate gene/miRNA	*Cattle*	[56]
20	49672680:49699785	*RIPK1*	Heat stress	GWAS	*Cattle*	[57]
20	49896021:49903711	*SERPINB9*	Heat stress	GWAS/review	*Ruminants*	[57,58]
20	49926117:49934850	*SERPINB1*	Heat stress	Gene expression	*Cattle*/*Buffaloes*	[46]
22	21182524:21232710	*HIF1AN* *†	Heat stress	Gene expression	*Cattle*	[58]

^1^ Genomic position in OAR assembly Ovis_aries_rambouillet.ARS-UI_Ramb_v2.0.112. ^2^ *GENES* with different symbols are highlighted as those associated with more than one indirect phenotype.

**Table 2:** Genes associated with indirect adaptation traits—including body size, amount of subcutaneous fat and lipid metabolism, immune system, and disease resistance—found in this study.

OAR	Position (bp) ^1^	Acronym ^2^	Evaluated Trait ^3^	Approaches in Literature	Species	Ref
1	97069164:97110990	*PHGDH*	Body weight	Gene expression	*Sheep*	[59]
1	101734837:101760081	*RORC*	Body fat deposition	Candidate gene	*Cattle*	[60]
1	101997411:102004301	*S100A11*	Wool	Gene expression	*Sheep*	[61]
1	215814932:216170153	*FNDC3B*	High altitude—Disease res.	Gene expression	*Cattle*	[53]
2	214941668:215134304	*IKZF2*	Adaptation to different environment	Selection signature	*Sheep*	[42]
2	231387211:231766014	*DNER*	High altitude—Disease res.	Gene expression	*Cattle*	[53]
2	233485205:233510593	*COPS7B* †	Fat tail vs. non-fat tail	Selection signature	*Sheep*	[62]
2	233423272:233484780	*PDE6D* †	Fat tail vs. non-fat tail	Selection signature	*Sheep*	[62]
3	2840050:2852203	*AGPAT2*	Body fat deposition (IMFM)	Gene expression	*Cattle*/*Sheep*	[63,64]
3	44148460:44206267	*PELI1* †‡	Immune response	GWAS	*Cattle*	[47]
3	147842320:147931790	*ABCD2*	Body fat deposition (IMFM)	Gene expression	*Sheep*	[65]
5	63118084:63132662	*MFAP3* †	Body fat deposition (IMFM)	GWAS	*Swine*	[66]
6	68539754:68567746	*SGCB*	Body weight (SM)	GWAS	*Sheep*/*cattle*	[67,68]
6	84931664:84970329	*YTHDC1*	Body fat deposition (SM)	Gene expression	*Sheep*	[69]
7	9452688:9601367	*SCAMP1*	Skeletal muscle	Gene expression	*Cattle*	[70]
7	45177062:45208358	*TPM1*	Body weight	Gene expression	*Sheep*	[71]
10	30475356:30502892	*USPL1*	Fat-tail vs. non-Fat-Tail	Selection signature	*Sheep*	[72]
15	44842835:44924038	*PTPRJ*	Adaptation high altitude	GWAS/Exome	*Goats*	[73]
15	50285158.:50365195	*RNF121* †	Body fat deposition	Gene expression	*Sheep*	[74]
15	44842835:44924038	*PTPRJ*	Adaptation high altitude	GWAS/Exome	*Goats*	[73]
19	21175321:21202741	*EDEM1*	UV resistance	GWAS	*Model O.*/*Human*	[75,76]
21	47447364:47471988	*SIRT3*	Body fat deposition	Candidate gene	*Cattle*	[77]
22	21025420:21041218	*SCD* †‡	Body fat deposition/(IMFM)	GWAS	*Sheep*/*cattle*	[78,79,80,81]
22	21182524:21232710	*HIF1AN* †	Skeletal muscle	Gene expression	*Cattle*	[82]
24	19178271:19216128	*LYRM1*	Body fat deposition	Candidate gene	*Cattle*	[83]

^1^ Genomic position in OAR assembly Ovis_aries_rambouillet.ARS-UI_Ramb_v2.0.112. ^2^ *GENES* with different symbols are highlighted as those associated with more than one indirect phenotype. ^3^ IMFM = intramuscular fatty acid metabolism; SM = Skeletal Muscle

**Table 3:** Genes found in our work and retrieved from the literature as being associated with milk traits. The reported genes fell within 250 kb upstream and downstream of *F_ST_* outlier SNPs.

OAR	Position (bp) ^1^	Gene Acronym ^2^	Evaluated Trait	Approaches in Literature	Species	Ref
1	215021462:215285520	*SPATA16*	Milk fat	GWAS	*cattle*	[84,85]
3	44148460:44206267	*PELI1* ‡	Udder/Immunity	GWAS	*cattle*	[86]
5	63268758:63495740	*GALNT10*	Milk fat	Candidate gene	*cattle*	[85]
6	36709616:36855827	*HERC3*	Milk Yield/Immune	Candidate gene	*cattle*/*sheep*	[86,87]
6	69212902:69607896	*SCFD2* ‡	Milk Yield/Beef vs. dairy	GWAS/selection signature	*cattle*/*goats*	[51,88,89]
22	20876528:20917538	*CHUK*	Milk yield	Selection signature	*sheep*	[90]
22	21025420:21041218	*SCD* ‡	Milk fat	Candidate gene/GWAS	*sheep*/*cattle*/*buffalo*	[79,80,91,92,93]
22	21141413:21172102	*SEC31B*	Milk fat	GWAS	*cattle*	[94]
24	19496885:19529560	*ZP2*	Milk Fat and Fatty Acid	Candidate gene	*sheep*	[95,96]
24	20438325:20506580	*EEF2K*	Milk yield	GWAS	*cattle*	[97]

^1^ Genomic position in OAR assembly Ovis_aries_rambouillet.ARS-UI_Ramb_v2.0.112. ^2^ *GENES* with different symbols are highlighted as those associated with more than one indirect phenotype.

**Table 4:** Gene enrichment analysis.

Source	GO	Term Name	AdjP	n	Intersection
MF	GO:0004857	Enzyme inhibitor activity	0.004	6	*SERPINE2 PDE6D SOCS3 SORL1 SERPINB9 SEPINB1*
MF	GO:0030060	L-malate dehydrogenase activity	0.005	2	*MDH1 PHGDH*
MF	GO:0140678	Molecular function inhibitor activity	0.017	6	*SERPINE2 PDE6D SOCS3 SORL1 SERPINB9 SERPINB1*
MF	GO:0016615	Malate dehydrogenase activity	0.025	2	*MDH1 PHGDH*
MF	GO:0019771	High-affinity IgG receptor activity	0.026	1	*FCGR1A*
MF	GO:0004866	Endopeptidase inhibitor activity	0.032	4	*SERPINE2 SORL1 SERPINB9 SERPINB1*
MF	GO:0030414	Peptidase inhibitor activity	0.036	4	*SERPINE2 SORL1 SERPINB9 SERPINB1*
MF	GO:0061135	Endopeptidase regulator activity	0.045	4	*SERPINE2 SORL1 SERPINB9 SERPINB1*
BP	GO:0051248	Negative regulation of protein metabolic process	0.049	7	*SERPINE2 SNX3 SOCS3 SORL1 PTPRJ SERPINB9 SERPINB1*
CC	GO:0031982	Vesicle	0.002	16	*FCGR1A S100A11 SEPINE2 DNER PDE6D UGP2 MDH1 MYO1G SNX3 SORL1 PTPRJ RIPK1 SEPINB9 SEPINB1 PHGDH AGPAT2*
CC	GO:1903561	Extracellular vesicle	0.005	11	*S100A11 SEPINE2 UGP2 MDH1 MYO1G SNX3 SORL1 PTPRJ SERPINB9 SEPINB1 PHGDH*
CC	GO:0043230	Extracellular organelle	0.005	11	*S100A11 SERPINE2 UGP2 MDH1 MYO1G SNX3 SORL1 PTPRJ SERPINB9 SEPINB1 PHGDH*
CC	GO:0065010	Extracellular membrane-bounded organelle	0.005	11	*S100A11 SERPINE2 UGP2 MDH1 MYO1G SNX3 SORL1 PTPRJ SERPINB9 SEPINB1 PHGDH*
CC	GO:0031410	Cytoplasmic vesicle	0.009	17	*FCGR1A S100A11 SERPINE2 DNER PDE6D SNX3 SORL1 PTPRJ RIPK1 SEPINB1 AGPAT2 SCAMP1 PPIB SPATA16 RAB8B SEC31B ZP2*
CC	GO:0097708	Intracellular vesicle	0.010	17	*FCGR1A S100A11 SERPINE2 DNER PDE6D SNX3 SORL1 PTPRJ RIPK1 SEPINB1 AGPAT2 SCAMP1 PPIB SPATA16 RAB8B SEC31B ZP2*
CC	GO:0070062	Extracellular exosome	0.029	10	*S100A11 UGP2 MDH1 MYO1G SNX3 SORL1 PTPRJ SEPINB9 SEPINB1 PHGDH*
CC	GO:0005769	Early endosome	0.033	5	*FCGR1A DNER SNX3 SORL1 SEPINB1*

## Data Availability

The raw data supporting the conclusions of this article will be made available by the authors on reasonable request. The elaborated *F_ST_* and meteorological data presented in the study are openly available in Supplementary File S3.

## Supplementary Materials

The following supporting information can be downloaded at: https://www.mdpi.com/article/10.3390/ani14243585/s1, Figure S1: (a) Distribution of weather stations; (b) 20-year average maximum and minimum temperature at weather stations sited at different altitudes; (c) Distribution of flocks and animal records; (d) Distribution of max temperatures. Supplementary Figure S2: (a) MDS plot of 825 samples on the first two principal coordinates in the groups (hot and cold); (b) MDS plot of 825 samples on the first two principal coordinates according to altitude of meteorological stations. Figure S3: STRING protein–protein interactions of 51 genes highlighted in this study. Table S1: Summary of the outlier *F_ST_* in the Sarda sheep genomes. Table S2: Outlier *F_ST_* in Sarda sheep genomes and relative statistics. Table S3: Gene retrieved in the 500 kb interval from *F_ST_* outlier signals. Table S4: The 51 genes retrieved in the 500 kb interval from *F_ST_* outlier signals associated with thermal stress, adaptation to environment, body size and fatness, immune system, and milk traits in the existing literature. Table S5: Output of Gprofiler2: gene enrichment analysis, GO, and gene intersection. Supplementary Files (.zip). Supplementary File S1: Script in Python to compute the *F_ST_* (Fst.py). Supplementary File S2: SAS script for computing LOESS-predicted *F_ST_* and control limits. Supplementary File S3: Flock, meteorological station, and temperature—complete raw *F_ST_* data.
