# Supplementary material for: Genomic Analysis of Sarda Sheep Raised at Diverse Temperatures Highlights Several Genes Involved in Adaptations to the Environment and Heat Stress Response
Source: Animals (Basel). 2024 Dec 12;14(24):3585. doi: 10.3390/ani14243585 (PMC11672698; doi:10.3390/ani14243585)
Supplement: Supplementary file 1 [file animals-14-03585-s001.zip › Supplementary Figures and Tables.zip!/Supplementary Figures.pdf]

a)

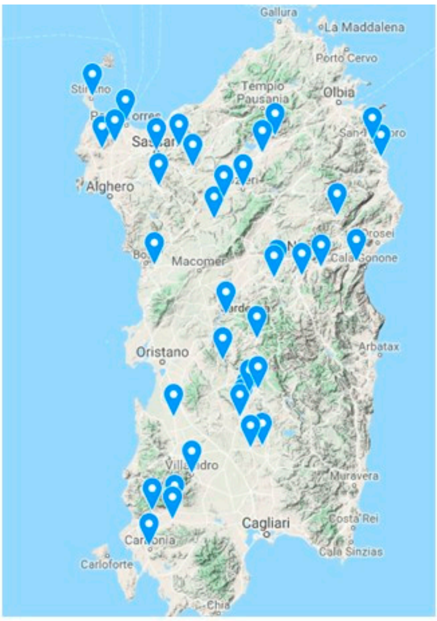

b)

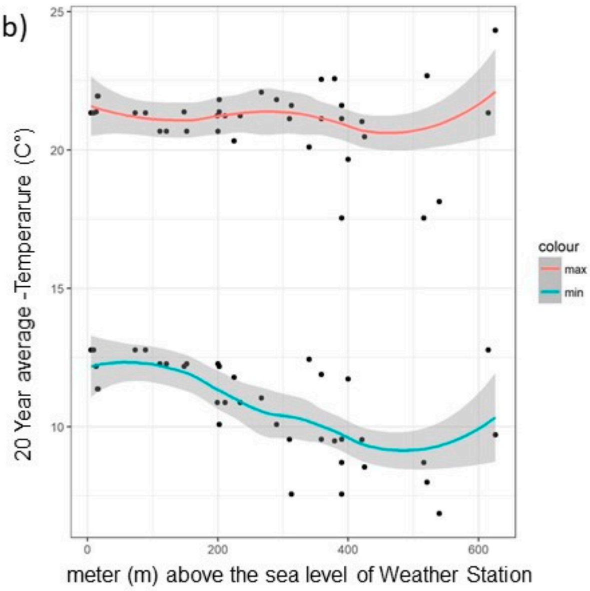

c)

Province	Flock	Records
Cagliari	14	284
Nuoro	14	248
Oristano	5	93
Sassari	13	200
**Total**	**46**	**825**

d)

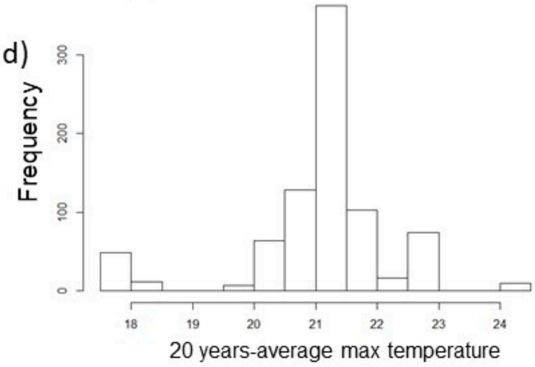

Supplementary Figure S1. a) Distribution of weather stations; b) 20-year average maximum and minimum temperatures at weather stations sited at different altitudes; c) distribution of flocks and animal records; d) distribution of max temperatures.

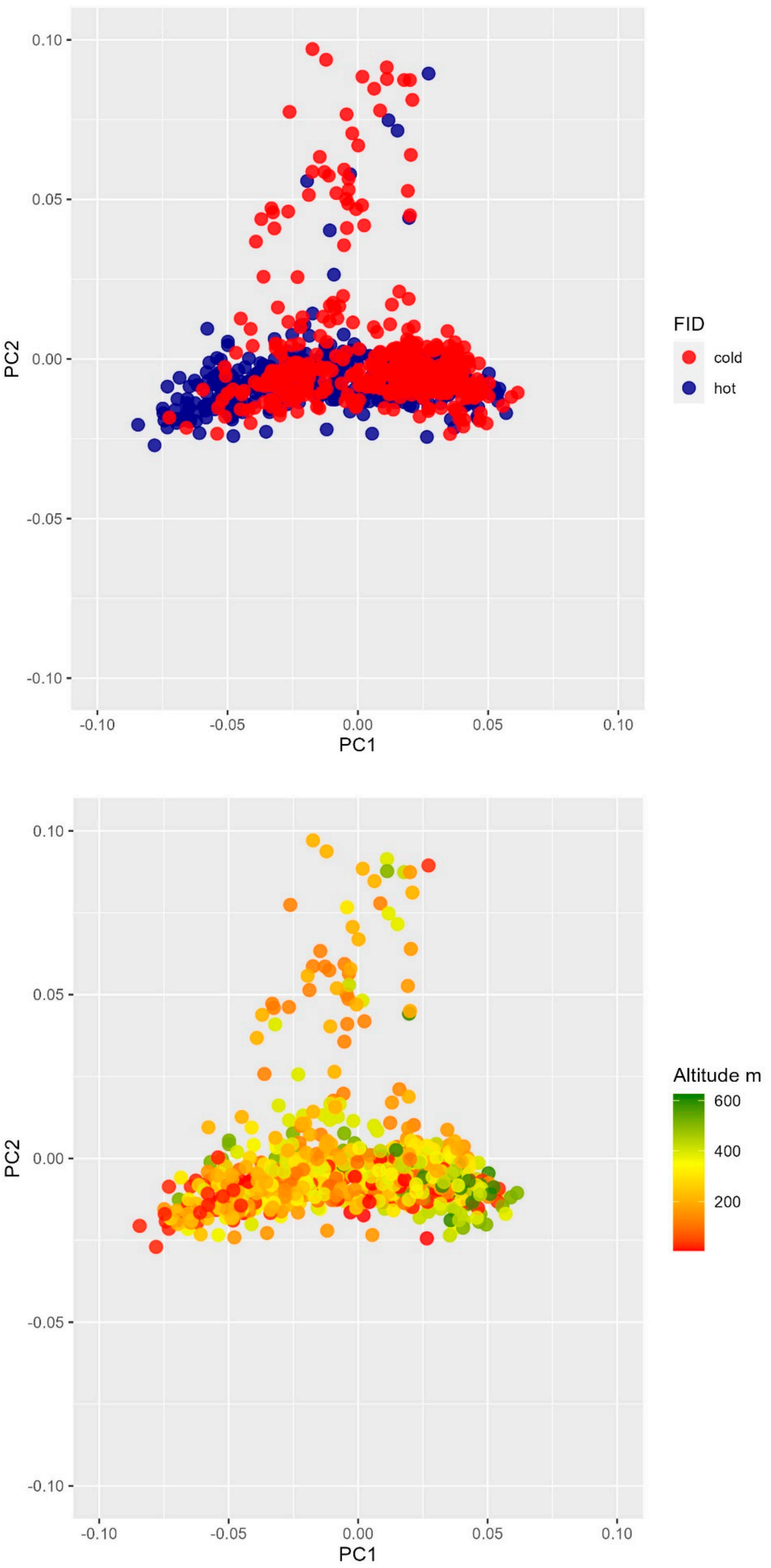

Supplementary Figure S2. **a)** MDS plot of 825 samples on the first 2 principal coordinates in the groups (hot and cold); b) MDS plot of 825 samples on the first 2 principal coordinates according to altitude of meteorological station.

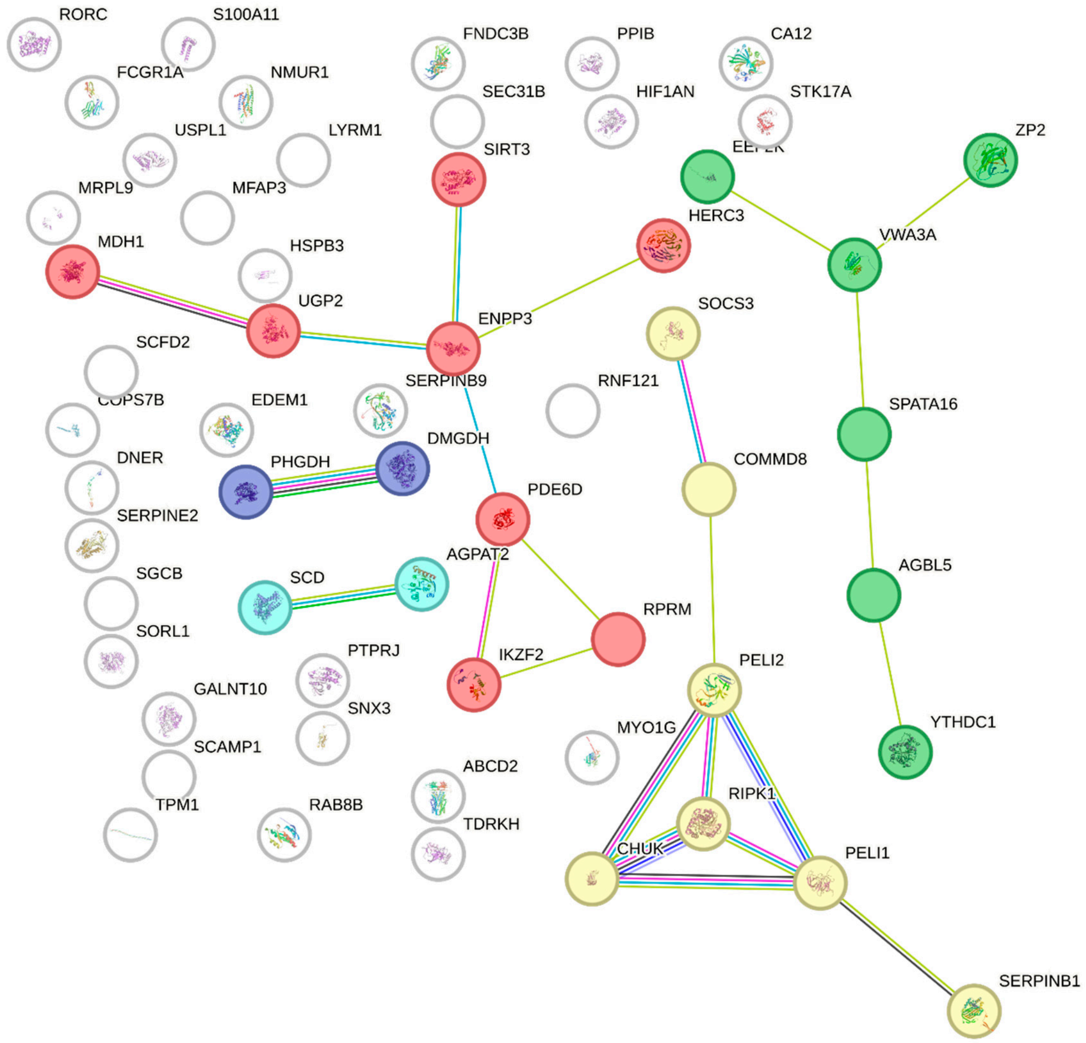

Supplementary Figure S3. String protein–protein interactions of 51 genes highlighted in this study.
